# Adherence to Clinical Practice Guidelines (CPG) management of dengue infection in adults (revised 2^nd^ edition)

**DOI:** 10.1371/journal.pone.0184559

**Published:** 2017-11-02

**Authors:** Marzilawati Abd.Rahman, Rafdzah Ahmad Zaki, Roza Sarimin, Mohd Izhar Ariff, Zailiza Suli, Maimunah Mahmud, Ker Hong Bee, Cecilia Anthonysamy, Azahirafairud Abdul Rahim, Balvinder Singh Gill, Shanti Rudra Deva, Ana Fizalinda Abdullah Sani, Erni Zurina Romli, Izzuna Mudla Mohamed Ghazali, Mohd. Aminuddin Mohd. Yusof, Nafisah Ahmad Lutfi, Shahril Effendi Shuib, Noormah Mohd Darus, Rugayah Bakri, ‘Abqariyah Yahya

**Affiliations:** 1 Department of Medicine, Hospital Kuala Lumpur, Kuala Lumpur, Malaysia; 2 Julius Centre University Malaya,Department of Social and Preventive Medicine, Faculty of Medicine,University Malaya, Kuala Lumpur, Malaysia; 3 Public Health Physician,Health Technology Assessment Section, Ministry of Health, Putrajaya, Malaysia; 4 Disease Control Division, Ministry Of Health, Putrajaya, Malaysia; 5 Family Medicine Specialist,Health Clinic, Ministry of Health, Jinjang, Malaysia; 6 Department of Medicine, Hospital Raja Permaisuri Bainun, Ipoh, Malaysia; 7 Department of Emergency Medicine, Hospital Serdang, Selangor, Malaysia; 8 Department of Anaesthesiology, Hospital Kuala Lumpur, Kuala Lumpur, Malaysia; Institute of Tropical Medicine (NEKKEN), Nagasaki University, JAPAN

## Abstract

The Malaysian Dengue Clinical Practice Guidelines (CPG) have been developed to provide evidence-based guidance in the management of dengue infections. The use of these guidelines is essential to ensure its recommendations are being practiced. However, the adherence to the guidelines for management of dengue (revised 2^nd^ edition) by healthcare providers still remains unknown. Therefore, the aim of this study was to evaluate the proportion among healthcare providers that adhere to this Dengue CPG. A retrospective cohort study of dengue cases registered from 1 January 2014 to 1 June 2015 was conducted in public hospitals and health clinics in Selangor, Putrajaya and Kuala Lumpur. Adherence to the CPG recommendations were recorded by reviewing patients’ case notes. Overall proportion of adherence in clinical components of the recommendation were (7.1 to 100.0% versus 7.7 to 73.8%) in history taking, (6.7 to 100.0% versus 12.3 to 60.0%) in physical examinations, (18.4 to 100.0% versus 23.1 to 83.2%) in assessment of warning signs, (0.6 to 100.0% versus 12.3 to 87.7%) in assessment of haemodynamic status, (60.0 to 100.0% versus 27.7 to 40.0%) in diagnosis, (46.6 to 80.0% versus 52.3%) in case notifications, (73.2 to 100.0% versus 89.2 to 96.9%) in performing specific laboratory investigations and (7.9 to 100.0% versus 21.5%) in monitoring, for outpatient versus inpatient, respectively. Adherence trends were demonstrated to be higher in hospital settings compared to outpatient settings. Adherence to this Dengue CPG varies widely with overall good clinical outcomes observed.

## Introduction

Dengue is the world’s most rapidly spreading and geographically widespread mosquito borne viral disease with an estimated 3.9 billion people in 128 countries currently at risk from dengue as reported by WHO [1]. Dengue is endemic in Southeast Asia, India, the Caribbean, Central and South America and Africa, with over 50 million new cases occurring annually [2]. In Malaysia, during the period 2002–2010, the incidence rate of dengue was 125 cases per 100,000 population, with an increasing trend observed in the annual number of dengue cases reported during the same period [2]. A total of 7,103 dengue cases were reported in year 2000, which increased to 46,171 cases in year 2010 [2]. Deaths due to dengue were also reported to have increased in the recent years. In 2013, a total 92 dengue deaths were reported in Malaysia, which increased to 215 deaths in 2014 and a further increase to 336 deaths in 2015 [3].

The failure to detect dengue cases with potential signs of deterioration (warning signs) may lead to the increased risk of dengue mortality. Several studies have showed that early detection of warning signs and appropriate fluid management contributes to good patient outcomes [4].

The aim of Clinical Practice Guidelines (CPG) Management of Dengue Infection in Adults (revised 2^nd^ edition) is to assist healthcare providers in making evidence-based decisions in the management of dengue infection in adults by improving the recognition and diagnosis of dengue cases and to provide appropriate care in order to reduce patient morbidity and mortality [5]. The Dengue CPG was the latest edition in Malaysia during the study period that summarized the best available evidence at the current time to provide a comprehensive set of recommendations to healthcare providers. The members of the Development Group (DG) consisted of relevant multidisciplinary specialties from Ministry of Health and Ministry of Higher Education namely family medicine specialist, emergency medicine specialist, general physician, infectious disease physicians and nursing sister. There was active involvement of multidisciplinary Review Committee during the development. Systematic literature search was carried out using the electronic databases. Statements and recommendations formulated were agreed by both DG and RC. This CPG is based largely on findings of systematic reviews, meta analysis and clinical trials with local practices taken into consideration. The development of this CPG was carried out in 2007 and this CPG was published on 2010. The number of cases in 2008 until 2010 was more than 40 000 cases in average per year and the number reduce to less than 22 000 cases per year in 2011 and 2012. Moreover, the mortality rate in 2008 until 2010 were 112, 88 and 134 dengue death reported whereas the dengue death rate reduce to less than 40 in year 2011 and 2012.The uptake of the guidelines by healthcare providers is essential to ensure these recommendations are practiced during patient care [6]. Healthcare providers’ adherence to evidenced-based management of dengue to ensure the delivery of appropriate and quality patient care have not been studied in Malaysia. Hence, healthcare providers’ adherence to the guideline for management of dengue (revised 2^nd^ edition) remains unknown.

Therefore, the aim of this study was to evaluate the adherence of this CPG among the healthcare providers. Specific objectives were to measure the proportion of patients managed based on the use of CPG Management of Dengue Infection in Adults (revised 2^nd^ edition) at different levels of care. Dengue management at government health clinics, hospital Emergency Department (ED) teams, medical teams and Intensive Care Unit (ICU) teams were evaluated to determine the association between adherence to the CPG by these teams managing dengue patients and the level of care and patient outcomes provided by these teams.

This study was registered with National Medical Research Register (NMMRID: 20233) and approved by the University of Malaya Medical Centre Ethical Committee (MEC ID: 201412–902).

## Materials and methods

### Target populations and sampling

A retrospective cohort study was conducted on registered dengue cases from 1 January 2014 until 1 June 2015 from e-Dengue registry, Ministry of Health Malaysia. The calculated sample size was 377 cases with an assumption of at least 50% of doctors adhered to the CPG and design effect of 1.0. Proportionate random sampling of registered dengue patients treated in public hospitals and health clinics in Selangor and Federal Territory (Kuala Lumpur & Putrajaya) provided by the Disease Control Division, Ministry of Health (MOH) was carried out. Only patients aged 12 years old and above were included in this study. All medical records were reviewed. Patients’ case notes were assessed based on the MOH CPG Management of Dengue Infection in Adults (revised 2^nd^ edition) recommendations.

### Data collection

The case report form (CRF) for evaluating adherence level of CPG was developed and validated. The CRF was separated into six sections (see S1 Appendix). In the first section, the baseline patient characteristics were collected which include age, gender and type of healthcare facility. From the second to fifth sections, data on first patient encounter to doctors were recorded for outpatient or health clinic, ED team, medical department’s team and ICU team. For each of these sections, data pertaining to history taking, diagnosis, laboratory investigations, early management and monitoring of dengue infection were collected. In the sixth section, data on individual patient outcomes in terms of mortality or morbidity, complication on hospital acquired infection, thrombophlebitis, patients’ need to be followed up after treatment and the management of dengue infection were recorded. Data collectors were the junior doctors and healthcare workers who underwent training by specialist family physicians, emergency physicians and internal medical physicians for CPG and details on how to acquire data from medical case notes. Training of data collectors occurred in two phases, in May 2015 and in September 2015. Data collection was conducted from June 2015 until March 2016.

### Adherence to clinical practice guideline

The latest edition of Dengue CPG in Malaysia used during the study period was ‘Clinical Practice Guidelines on Management of Dengue Infection in Adults (revised 2^nd^ edition, 2010), which is the revised version of Dengue CPG (2^nd^ edition, 2008). Based on these guidelines, data collections were divided into four sections which were the first encounter at the outpatient clinic, ED, medical team and ICU team. Presence of documentation in patient’s clinical notes as recommended by CPG was defined as adherence to CPG.

### Data analysis

All CRF were checked and verified by trained personnel. Verified data were stored in MS Access database. Quality control by trainer was performed by taking 10% of the data from the database and comparing it with the physical data to assure consistency of the data. Descriptive statistics related to each exposure variable were tabulated displaying its frequency and percentage. Statistical tests were conducted at 5% significance level and data analysis was performed using IBM SPSS Statistical Software Version 22. The association between categorical variables was measured using Chi-square statistics. The continuous variables were presented by mean, standard deviation, median, minimum and maximum values and measured using appropriate statistical analysis depending on the type of distribution. Results were compared between weighted and unweighted analysis. Since the results were comparable, the unweighted result is presented here.

## Results and discussion

### Characteristics of patients

From 377 cases eligible for the study, only 326 cases were included. A total of 51 cases were excluded due to age less than 12 years, cases not from 1 January 2014 until 1 June 2015, and missing records. From the 326 cases included in the study, a total of 261 cases (80%), were from hospitals and 65 (20%) were from outpatient settings.

**Fig 1:**
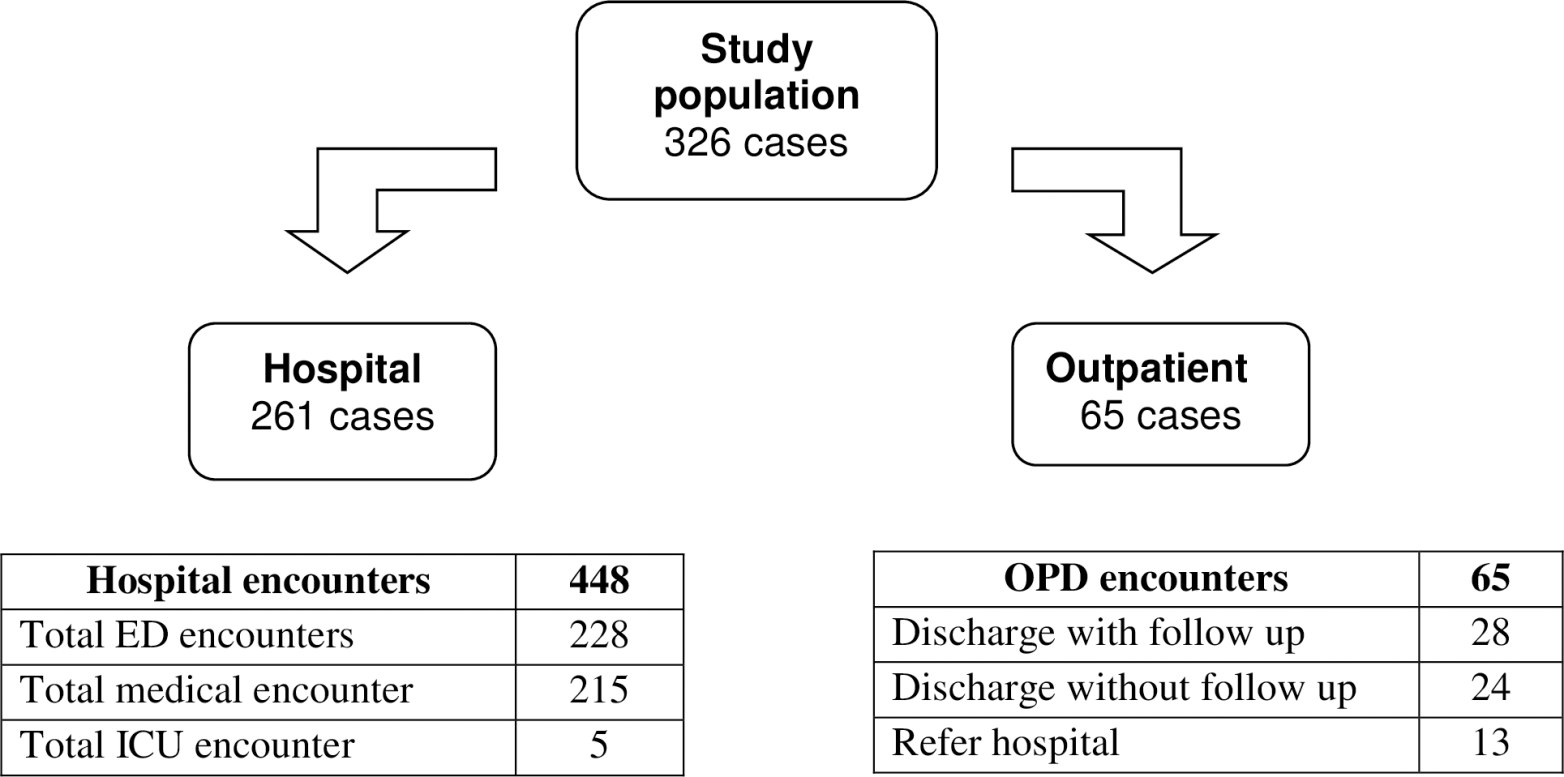
Dengue assessment encounters.

Fig 1 details 513 encounters derived from the 326 cases. A total of 65 encounters were from outpatient settings while 448 encounters were from hospital settings. Within hospital encounters, 228 encounters were from ED, 215 encounters from Medical teams and 5 encounters were from ICU teams. From 65 outpatient encounters, 13 cases needed to be referred to hospital, 28 cases were discharged with follow up, and 24 cases were discharged without follow up.

A total of 196 cases (60.1%) were male and 130 cases (39.9%) were female. Median age of patients was 26 years, with only 2.8% (9 cases) aged more than 65 years. There were 14 cases (4.3%) with previous history of dengue and five cases (1.2%) were pregnant women. Average length of warded was two days. However, maximum length of warded was 40 days. Underlying co-morbidities were found in 25 cases (7.7%), of which 5.2% (17) diabetes mellitus, 1.5% (5) hypertension, 0.3% (1) ischemic heart disease and 0.6% (2) morbid obesity. There were 53.7% of cases with health clinic as first place of consultation and 46.3% with ED as the first place of consultation for dengue infection (Table 1).

**Table 1:** Demography and baseline information.

Patients characteristics	Total, N (%) 326 Cases
**Gender**	
Male	196 (60.1)
Female	130 (39.9)
Elderly (>65 years old)	5 (1.5)
Previous Dengue History	14 (4.3)
Pregnancy	4 (1.2)
**Co morbidities**	25 (7.7)
DM	17 (5.2)
HPT	5 (1.5)
IHD	1 (0.3)
Morbid Obesity	2 (0.6)
**Place of First Consultation**	
Primary care	175 (53.7)
Emergency department	151 (46.3)
Age, years Median (min, Max)	26 (13,77),N = 322
Length of hospitalization, days Median (Min,Max)	2 (0,40) N = 220

### Adherence to the dengue CPG

Mandatory notification of dengue under the Prevention and Control of Infectious Diseases Act 1988 (Act 342) to the nearest District Health Office as stated in the CPG should be done within 24 hours of diagnosis. Notifications were documented in four (80.0%) of the ICU encounters, 165 (76.7%) encounters from medical teams, 34 (52.3%) encounters and 152 (46.6%) encounters respectively from outpatient clinics and emergency departments (Table 2).

**Table 2:** Disease notification.

Clinical Documentation	Outpatient (N = 65), n (%)	Emergency Department (N = 228), n (%)	Medical team (N = 215),n (%)	ICU team (N = 5), n (%)
Notification within 24 hours from diagnosis	34 (52.3)	152 (46.6)	165 (76.7)	4 (80)

In terms of blood investigations; Full Blood count (FBC), Haematocrit (HCT) and Dengue Serology in dengue patients were also recommended in each encounter for dengue cases. In outpatient settings, 63 (96.9%) encounters documented FBC results and only 58 (89.2%) encounters documented patient’s HCT levels (Table 3). FBC and HCT were documented; five (100.0%) encounters in ICU teams, 213 (99.1%) in medical teams and 220 (96.5%) encounters in emergency department settings.

**Table 3:** Documented investigation (outpatient).

Documented investigation	Outpatient, N = 65, n(%)
Full blood count (FBC)	63 (96.9)
Haematocrit (HCT)	58 (89.2)

As for documenting investigations at inpatient settings, both FBC and HCT were documented; five (100.0%) encounters in ICU teams, 213 (99.1%) in medical teams and 220 (96.5%) encounters in emergency department settings. Dengue confirmation test were documented in five (100.0%) encounters by ICU teams, 187 (87.0%) encounters in medical teams and 167 (73.2%) in emergency departments (Table 4).

**Table 4:** Documented investigation (inpatient).

Clinical Documentation	Emergency department N = 228, n (%)	Medical team N = 215, n (%)	ICU team N = 5, n (%)
**FBC & HCT**	220/228(96.5)	213/215(99.1)	5/5(100)
Febrile	160/220 (72.7)	120/213 (56.3)	1/5 (20)
Critical	55/220 (25)	89/213 (41.8)	4/5 (80)
Recovery	5/220 (2.3)	4/213 (1.9)	0
**Dengue Serology**	167/228(73.2)	187/215(87)	5/5(100)
Febrile	121/167 (72.5)	107/187 (57.2)	1/5 (20)
Critical	42/167 (25.1)	79/187(42.2)	4/5 (80)
Recovery	4/167 (2.4)	1/187(0.5)	0

Overall 265 (81.3%) cases had either Dengue antibody IgG, IgM or NS1Ag being performed. There were 243 (91.7%) cases that were reported as either one of the confirmation test positive (Table 5).

**Table 5:** Documented diagnostic test.

Dengue Tests: Dengue Antigen Antibody test performed (IgG, IgM, NS1Ag)	Total cases n (%)
Any Test Done	265/326 (81.3)
Any Positive Test	243/265 (91.7)

Documentation of dengue case history is a requirement in the CPG. Eight clinical variables are recommended in the CPG; (1) date of onset of fever/illness, (2) Oral intake, (3) Diarrhoea (4) Bleeding (5) Change in Mental state/ seizures/ dizziness, (6) Urine frequency, (7) Urine volume, and (8) Time of last voiding. In the history taking section, the highest documentation was ‘Bleeding’, 100% documentation by the ICU teams followed by 89.8% in medical teams. Lowest percentage of documentation was information on ‘Time of last voiding’ 7.7% in outpatient departments and 10.9% in ED settings. ‘Date of onset of fever/illness’ was documented ranged from 20% to 73.8% across 4 areas. ‘Oral intake history’ was noted to be documented in 40.0% to 62.3%, while ‘Change in mental state/seizures/dizziness’ had a range of 26.8% to 40% documentation. ‘Urine volume’ was documented in range of 10.1% to 60% (Table 6).

**Table 6:** Documented history.

Clinical variables	Outpatient (N = 65), n (%)	Emergency department (N = 228), n (%)	Medical team (N = 215), n (%)	ICU team (N = 5), n (%)
Date of onset of fever/illness	48 (73.8)	115 (50.4)	158 (73.5)	1 (20)
Oral intake	37 (56.9)	142 (62.3)	131 (60.9)	2 (40)
Diarrhoea	39 (60.0)	169 (74.1)	181 (84.2)	3 (60)
Bleeding	31 (47.7)	196 (85.9)	193 (89.8)	5 (100)
Change in mental state/seizure/dizziness	18 (27.7)	61 (26.8)	73 (34.0)	2 (40)
Urine frequency	8 (12.3)	53 (23.2)	46 (21.4)	2 (40)
Urine volume	15 (23.1)	23 (10.1)	52 (24.2)	3 (60)
Time of last voiding	5 (7.7)	25 (10.9)	52 (24.2)	3 (60)

CPG requires assessment of warning signs which include ‘abdominal pain’ or ‘abdominal tenderness’, ‘persistent vomiting’, ‘clinical fluid accumulation’, ‘mucosal bleed’, ‘restlessness or lethargy’,’ tender enlarged liver’ and laboratory test trend of an increase in HCT and a decrease in platelet counts. Documentation of warning signs was below 83.1% in outpatient setting, while abdominal pain and persistent vomiting were documented in 69.2%. Adherence to documentation of these warning signs was better in ED and medical departments with more than 95.0% adherence. Abdominal pain or tenderness and HCT were documented at 100% in ICU, followed by clinical fluid accumulation 96.1% in ED. On the contrary, tender enlarged liver was poorly documented which was less than 40.0% in outpatient and ED settings. Clinical fluid accumulation was poorly documented in outpatient with 50.8% adherence compared to 96.1% and 94.4% respectively in ED and medical teams. Restlessness or lethargy was only documented between 20.0 to 38.5% in all settings. Mucosal bleed were documented in less than 50% in outpatient settings, but more than 70% in ED and medical departments (Table 7 and Table 8).

**Table 7:** Documented assessment for warning signs and physical examination and hemodynamic status.

Clinical variables	Outpatient (N = 65), n (%)	Emergency department (N = 228), n (%)	Medical team (N = 215),n (%)	ICU team (N = 5), n (%)
**Assessment for warning signs**				
Abdominal pain or tenderness	45 (69.2)	217 (95.2)	206 (95.8)	5 (100)
Persistent vomiting	45 (69.2)	217 (95.2)	204 (94.9)	4 (80)
Clinical fluid accumulation (pleural effusion, ascites)	33 (50.8)	219 (96.1)	203 (94.4)	4 (80)
Mucosal bleed	32 (49.2)	166 (72.8)	170 (79.1)	3 (60)
Restlessness or lethargy	25 (38.5)	60 (26.3)	75 (34.9)	1 (20)
Tender enlarged liver	15 (23.1)	86 (37.7)	126 (58.6)	3 (60)
Laboratory: Increase in HCT concurrent with rapid decrease in platelet	54 (83.1)	197 (86.4)	173 (80.5)	5 (100)
**Physical examination**				
Assess mental state and Glasgow Coma Scale (GCS) score	34 (52.3)	211 (92.5)	205 (95.3)	5 (100)
Assess hydration status	39 (60.0)	187 (82.0)	174 (80.9)	5 (100)
Look out for tachypnoea/ acidotic breathing	36 (55.4)	183 (80.3)	168 (78.1)	3 (60)
Look out for pleural effusion	38 (58.5)	220 (96.5)	202 (94.0)	5 (100)
Examine for bleeding manifestation	35 (53.8)	73 (32.0)	85 (39.5)	2 (40)
Check for abdominal tenderness	34 (52.3)	214 (93.9)	209 (97.2)	5 (100)
Check for hepatomegaly	8 (12.3)	54 (23.7)	109 (50.7)	1 (20)
Check for ascites	15 (23.1)	22 (9.7)	35 (16.3)	1 (20)

**Table 8:** Documented assessment for hemodynamic status.

Clinical variables	Outpatient (N = 65), n (%)	Emergency department (N = 228), n (%)	Medical team (N = 215),n (%)	ICU team (N = 5), n (%)
**Haemodynamic status**				
Skin colour	22 (32.3)	122 (53.5)	82 (38.1)	2 (40)
Cold/ warm extremities	21 (32.3)	172 (75.4)	192 (89.3)	5 (100)
Capillary filling time (normal <2 seconds)	28 (43.1)	205 (89.9)	208 (96.7)	5 (100)
Pulse rate	56 (86.2)	198 (86.8)	211 (98.1)	5 (100)
Pulse volume	11 (16.9)	190 (83.3)	197 (91.6)	5 (100)
Blood pressure	57 (87.7)	200 (87.7)	212 (98.6)	5 (100)
Pulse pressure	8 (12.3)	2 (0.9)	8 (3.7)	5 (100)
**Phase of illness**	26(40.0)	199(87.3)	204(94.9)	3(60)
Febrile	18 (45.0)	137 (68.8)	100 (46.5)	2 (66.7)
Deferversence/Critical	6 (23.1)	58 (29.1)	101 (49.5)	1 (33.3)
Recovery	2 (7.7)	4 (2.0)	3 (1.5)	0
**Dengue diagnosis**	18(27.7)	206(90.4)	205(95.3)	5(100)
Dengue without warning sign	8 (44.4)	57 (27.7)	35 (17.1)	0
Dengue with warning sign	10 (55.6)	148 (71.9)	168 (82.0)	1 (20)
Severe plasma leakage	0 (0)	1 (0.5)	2 (0.1)	3 (60)
Severe bleeding	0 (0)	0	0	3 (60)
Severe organ impairment	0 (0)	0	0	2 (40)

Two components that are recommended in the CPG are physical examination and assessment of haemodynamic status. It is mandatory to assess the mental state, hydration status, signs of tachypnoea, acidotic breathing, pleural effusion, bleeding manifestations, hepatomegaly and ascites during the physical examination. Assessment of haemodynamic status include skin colour, cold/warm extremities, capillary filling time, pulse rate, pulse volume, blood pressure and pulse pressure. As for the physical examination section, assessment of mental state, Glasgow Coma Scale score, assessment of hydration status, pleural effusion and abdominal tenderness was the highest documented which were 100% in ICU team and more than 80.0% from other departments. Mental state examination was done in more than 92.0% of cases in ED and in hospital settings, while it was documented in 52.3% in outpatient clinics. There was more than 50.0% adherence for examination of abdominal tenderness, however the adherence for examination of hepatomegaly and ascites were much lower across all departments. Blood pressure and pulse rates were consistently documented in more than 85.0% of all encounters. In the assessment of haemodynamic status, the entire variable was 100.0% documented in ICU except skin colour which only 40.0% documented. Among medical teams, blood pressure (98.6%), pulse rate (98.1%), capillary filling time (96.7%) and pulse volume (91.6%) was documented. These were among the highest documented and adhered. The lowest adherence in this section was pulse pressure (0.9%) in ED (Table 8).

Malaysia’s Dengue CPG, recommends that clinicians should be able to determine the diagnosis, disease staging and severity assessment based on the evaluation of history, physical examination, and FBC with HCT. Our study showed that in the outpatient clinic, 27.7% had documented complete dengue diagnosis (with or without warning signs), and 40.0% documented phase of dengue illness. Medical and emergency departments documented the highest adherence in terms of recording phase of illness and complete dengue diagnosis. ICU was noted to have 100.0% adherence for documented complete dengue diagnosis with two third of patients in febrile phase. Majority of patients in outpatient (55.6%), emergency (71.9%) and medical department (82.0%) had documentation of dengue with warning signs. All the patients in ICU had complete dengue diagnosis documented (Table 8).

The dengue CPG recommends that patients who do not need hospital admission, needs to be followed up at least daily with a Home Care Advice Leaflet until patients become afebrile for 24 to 48 hours without antipyretics. In the case where admission is indicated, patients need to be optimised pre-transfer from the clinic. Pre-transfer information needs to be communicated to the receiving team. Our study showed, all outpatient dengue patients had a median of two days clinic review (1–8 days), with 21.5% documented given home care advice leaflet for dengue patients. Of the 13 outpatients that needed hospital referral, 61.5% had management optimised prior to the transfer but only 30.8% was informed to ED/medical pre-transfer (Table 9).

**Table 9:** Documented plan of management.

Clinical documentation	Days / n(%)	
Number of daily review, median(min-max), N = 65	2 (1–8) days	
Home Care Advice Leaflet for Dengue Patients given, N = 65	**Yes**	**No**
	14 (21.5%)	51 (78.5%)
**Prerequisites for transfer**		
Patient was *optimized pretransfer, N = 13	8 (61.5%)	5 (38.5%)
ED/Medical was informed pretransfer, N = 13	4 (30.8%)	9 (69.2%)
Adequate information includes fluid chart, monitoring chart and investigation result given, N = 13	6 (46.2%)	7 (53.8%)

*Optimize: Complete resuscitation

Parameters and frequency of monitoring according to different phases of dengue illness are stated in the CPG. These parameters include ‘pink/cyanosis’, ‘extremities (cold/warm)’, ‘capillary refill time’, ‘pulse volume’, ‘pulse rate’, ‘blood pressure’, ‘respiratory rate’, ‘oxygen saturation’, ‘warning sign assessment’ and ‘urine output’. ICU teams adhered to 100% documentation of all monitoring parameters. Medical teams documented more than 85% of parameters except 43.3% in ‘pink/cyanosis’ and 18.1% in pulse pressure. Documentations of monitoring parameters varied in emergency setting. More than 80% were documented in ‘capillary refill time’, ‘pulse volume’, ‘pulse rate’, ‘blood pressure’, and ‘warning sign assessment’. More than 50.0% documentations were observed in ‘pink/cyanosis’, extremities ‘cold/warm’, ‘respiratory rate ‘and ‘oxygen saturation’. Poor documentations were noted in ‘urine output’ of 39.9% and ‘pulse pressure’ of 7.1% respectively (Table 10).

**Table 10:** Documented patient monitoring.

Clinical Documentation	Emergency department (N = 228), n (%)	Medical team (N = 215), n (%)	ICU team (N = 5), n (%)
**Pink/ cyanosis**	119/228(52.2)	93/215(43.3)	5/5(100)
Febrile	84/119 (70.6)	53/93(56.9)	1/5(20.0)
Critical	34/119 (28.6)	38/93(40.9)	4/5(80.0)
Recovery	1/119 (0.8)	2/93(2.2)	0
**Extremities (cold/warm)**	169/228(74)	186/215(86.5)	5/5(100)
Febrile	126/169(74.6)	107/186(57.5)	1/5(20.0)
Critical	41/169 (24.3)	75/186(40.3)	4/5(80.0)
Recovery	2/169 (1.2)	4/186(2.2)	0
**Capillary refill time**	195/228(85.5)	195/215(90.7)	5/5(100)
Febrile	145/195(74.4)	111/195(56.9)	1/5(20.0)
Critical	46/195(23.6)	80/195(41.0)	4/5(80.0)
Recovery	4/195(2.1)	4/195(2.1)	0
**Pulse Volume**	189/228(82.9)	187/215(87.0)	5/5(100)
Febrile	138/189(73.0)	106/187(56.7)	1/5(20.0)
Critical	47/189(24.9)	77/187(41.2)	4/5(80.0)
Recovery	4/189(2.1)	4/187(2.1)	0
**Pulse rate**	191/228(83.8)	211/215(98.1)	5/5(100)
Febrile	140/191(73.3)	119/211(56.4)	1/5(20.0)
Critical	48/191(25.1)	89/211(42.2)	4/5(80.0)
Recovery	3/191(1.6)	3/211(1.4)	0
**Blood pressure**	191/228(83.8)	212/215(98.6)	5/5(100)
Febrile	140/191(73.3)	120/212(56.6)	1/5(20.0)
Critical	48/191(25.1)	88/212(41.5)	4/5(80.0)
Recovery	3/191(1.6)	4/212(1.9)	0
**Pulse pressure**	18/228(7.9)	39/215(18.1)	5/5(100)
Febrile	11/18(61.1)	22/39(56.4)	1/5(20.0)
Critical	6/18(33.3)	16/39(41.0)	4/5(80.0)
Recovery	1/18(5.6)	1/39(2.6)	0
**Respiratory rate**	162/228(71.1)	188/215(87.4)	5/5(100)
Febrile	122/162(75.3)	110/188(58.5)	1/5(20.0)
Critical	39/162(24.1)	76/188(40.4)	4/5(80.0)
Recovery	1/162(0.6)	2/188(1.1)	0
**SpO2**	168/228(73.7)	185/215(86.0)	5/5(100)
Febrile	122/168(72.6)	107/185(57.8)	1/5(20.0)
Critical	44/168(26.2)	75/185(40.5)	4/5(80.0)
Recovery	2/168(1.2)	3/185(1.6)	0
**Warning sign assessment**	185/228(81.1)	197/215(91.6)	5/5(100)
Febrile	133/185(71.9)	113/197(57.4)	1/5(20.0)
Critical	48/185(25.9)	80/197(40.6)	4/5(80.0)
Recovery	4/185(2.2)	4/197(2.0)	0
**Urine output**	91/228(39.9)	197/215(91.6)	5/5(100)
Febrile	59/91(64.8)	114/197(57.9)	1/5(20.0)
Critical	31/91(34.1)	80/197(40.6)	4/5(80.0)
Recovery	1/91(1.1)	3/197(1.5)	0

The overall proportions of adherence for the eight components of the CPG; history, physical examination, assessment for warning signs, hemodynamic status, diagnosis, notification, investigation and monitoring were varied across all setting. In health clinics, high documentation was seen in ‘investigation’ with proportion range of 89.2% to 96.9%. A wide range was observed in the proportion of adherence for assessment of hemodynamic status, 12.3% to 87.7% and assessment for warning sign, 23.1% to 83.3%. In the history and physical examination component, proportion of adherence was lower with a range of 7.7% to 73.8% and 12.3% to 60.0% respectively. The lowest adherence in health clinics was seen in ‘diagnosis’ (27.7% to 40.0%), dengue notification (52.3%) and monitoring / home base card (21.5%). In hospital settings, the highest proportion of adherence was seen in dengue diagnosis with 60.0% to 100.0%, and dengue investigations with 73.2% to 100.0%. A wider range in the proportion of adherence was seen in dengue history (7.1% to 100%), physical examination (6.7% to 100.0%), assessment of warning signs (18.4% to 100%), assessment of haemodynamic status (0.6% to 100.0%) and in monitoring of dengue (7.9% to 100.0%). Notification of dengue was not up to 100%, with a range of proportion of adherence at only 46.6% to 80% (Table 11).

**Table 11:** Overall proportions of documentation (adherence).

Components	Proportion of Adherence (%)	
	Health clinic	Hospital
History	7.7–73.8	7.1–100.0
Physical Examination	12.3–60.0	6.7–100.0
Assessment for warning Signs	23.1–83.1	18.4–100.0
Assess hemodynamic status	12.3–87.7	0.6–100.0
Diagnosis	27.7–40.0	60–100.0
Notification	52.3	46.6–80.0
Investigation	89.2–96.9	73.2–100.0
Monitoring/Home Based Card	21.5	7.9–100.0

No death was reported in our study population. In health clinics, 20.0% (13) were referred to hospitals. 43.1% (28) of dengue patients needed follow up and 36.9% (24) did not need follow up. In hospital settings, five cases (1.9%) were admitted to ICU with one patient needing non-invasive ventilation while others needing invasive ventilation. There was 67.2% (175) discharged from hospital and needed follow up and 31.6% (83) discharged without follow up. Complications that occurred during hospitalisation were thrombophlebitis four cases (< 2%), hospital acquired pneumonia and other type of complications (unspecified) were respectively one case (<1%). Mean length of hospitalisation was 3.4 days (Table 12).

**Table 12:** Overall outcomes.

Section	Outcome	n (%)
Outpatient (n = 65)	Follow up	28(43.1)
	No follow up	24(36.9)
	Refer to hospital	13(20.0)
	Death	0
Hospital (n = 261)	Discharge with follow up	175(67.2)
	Discharge without follow up	83(31.6)
	ICU admission	5(0.7)
	Non-Invasive ventilation	1(0.4)
	Invasive ventilation	1(0.4)
	Thrombophlebitis	4(1.6)
	Fluid overload	0
	Hospital Acquired Pneumonia	1(0.4)
	Other complications	1(0.4)
	Death	0
	Length of hospital stay (mean)	3.4 days

During the period of the study, the latest edition of Dengue CPG in Malaysia was Clinical Practice Guidelines on Management of Dengue Infection in Adults (revised 2^nd^ edition, 2010). This CPG is the revised version of previous CPG (2nd Edition, 2008). The main objective of this CPG is to provide evidence-based guidance in the management of dengue infection in adult patients, improve recognition and diagnosis of the dengue cases and provide appropriate care to the patients. However adherence in clinical practice has not been studied. The aim of this study was to assess the degree of healthcare providers’ adherence to the Malaysia’s Dengue CPG in Ministry of Health facilities in Selangor and Federal Territory (Kuala Lumpur & Putrajaya), Malaysia. Adherence to CPG will reduce variation in practice on the management of dengue and hence appropriate management and quality patient care can be delivered.

Male preponderance in our study was similar to that reported in other dengue studies [7, 8]. Majority of the cases belonged to the young age group. Chew et al. reported highly endemic dengue in Malaysia especially among the 20 to 29 age group that was vulnerable to dengue infection [9]. This finding is also similar to another locally reported study by Mohd-Zaki et al. and also reported in studies from India, Pakistan and Saudi Arabia [2, 7,10, 11]. Young male adults were affected mainly because they are involved in outdoor activities more than the females which allowed them to be exposed to *Aedes* mosquitoes. Our study captured a small percentage (1.5%) of elderly with dengue infection. We defined elderly as aged 65 and above. Two local studies have shown less than 6% of dengue patients were from elderly population (aged 60 years and above) [9, 12]. A study by Rowe et al. reported 4.4% of elderly aged 60 years and above with dengue infection [13]. This is possibly as a result of less outdoor exposure. However, diagnosing dengue in this group is challenging as the presentation can be atypical.

It is important to assess co-morbidities and pregnancy status in managing dengue as these populations are more vulnerable for complications. In this study, only a small percentage had co-existing illness which included diabetes mellitus, hypertension and ischemic heart disease and the findings were similar to the study conducted by Rowe et al. [13]. This could be because dengue infection is more common in younger age group with lower prevalence of non-communicable diseases. Length of hospitalisation (LOS) for dengue can be varied depending on phase of dengue during admission, co-existing illness and severity of dengue. Khalil et al. reported mean of LOS was 3.46 ±3.45 days whereas Rowe et al. reported median of LOS was 4 days [13, 14].

The results of this study showed that there was a wide range of adherence to dengue CPG depending on type of facility and sections of the CPG. In hospital setting, the highest proportion of adherence was seen in ICU team, followed by medical team. This is probably due to severe dengue patient being monitored very closely in ICU. In medical wards, dengue cases are managed in dedicated dengue wards with trained dedicated staff. The lowest proportion of adherence in hospital setting was seen in emergency departments. This may possibly be due to the high workload of patients and short hospitalization in the area prior to assessment or transfer to medical ward. In health clinics, overall lower proportion of adherence was observed compared to hospital setting. This may possibly be due to similar reasons seen in emergency department setting and cases may probably be from the early phase of dengue illness. In ED and health clinics, the patients may present initially with undifferentiated fever, with symptoms suggestive of URTI or with high total white counts where dengue fever was probably not suspected. Often the dengue diagnosis was made following daily follow up where dengue features became clearer [15].

There was more than 80% adherence observed in blood investigation FBC and dengue serology testing in health clinics. This study identified lower adherence, about 20% adherence in giving home based card to outpatient for management of dengue fever. This figure may be underestimated due to poor documentation.

Overall proportions of adherence in dengue history taking recommended by CPG demonstrated lower adherence (about 20% or less) in urinary frequency, volume, time of last voiding as well as change in mental state. There is low adherence in documentation of pulse pressure in assessment of hemodynamic status, despite high adherence recorded in all sections of patients’ monitoring, except urine output specifically in outpatient setting. This result indicates that more compliance needs to be emphasised to healthcare workers in this area. Utilization and compliance in using current gazetted dengue monitoring chart in inpatient setting could contribute to good adherence in patients’ monitoring section. The outpatient dengue clerking sheet and home based card should be fully utilised to improve documentation and adherence.

The overall adherence in abdominal assessment to look for tender hepatomegaly or ascites was low. An appropriate examination room is required to ensure privacy and proper examination to be carried out. Therefore, to enhance adherence in this area, having an appropriate setting is crucial. However, high number of patients who present to outpatient setting may probably hinder doctors to make proper abdominal examinations due to consultation time constraints. Another factor for low adherence was the possibility that examination was done but not documented.

To the best of our knowledge, this is the first study that evaluates adherence to CPG using objective measurements in the local healthcare providers. This study focused on key recommendations in assessing adherence. It provided useful data that reflects the degree of implementation of the guidelines in clinical practice.

Limitation in this study includes information bias such as incompleteness of medical records and misclassification of cases by the data collectors. Secondly, in the present study, case notes documentations were used as a sole measure of adherence. Ideally healthcare providers’ compliance should be examined by using a more composite indicator that would evaluate all sections in dengue CPG. Thirdly, the current study only evaluated the level of adherence to certain parts of CPG namely laboratory investigations, assessments of dengue and disease monitoring of dengue for inpatient and outpatient setting in Selangor and Federal Territory public hospital and clinics which may not represent overall situation in non-participating hospitals and private hospitals.

In this study, documentations have been used as an equivalent to adherence to the CPG. Documentations may be affected by several factors such as time limitation, high volume of patients and lack of awareness. Retrospective examination of documentations may not reflect the true adherence.

## Conclusions

In summary, this study showed that adherence of health care providers to Dengue CPG varies widely based on documentations. Overall statement about adherence is impossible with a mixture of high and low adherence in certain parts of the CPG. However, good clinical outcomes were observed with current proportion of adherence.

This study suggested several recommendations. Awareness on the importance of complete record documentation need to be emphasized to healthcare providers in all healthcare settings, since lower proportion of adherence may possibly be due to poor documentation of medical records by health care workers. A complete and standard dengue clerking and monitoring sheet could be utilized to facilitate guidelines adherence in all setting. Further studies on quality, such as prospective evaluation and clinical audit may be conducted in the future to ascertain true proportion of adherence among healthcare providers in this country.

## Supporting information

S1 Appendix Pro-forma of Malaysian CPG adherence to management of dengue infection in adults. (PDF)

## References

[1] World Health Organization [Internet]. Geneva: WHO; c2017. Dengue and severe dengue. Available from: http://www.who.int/mediacentre/factsheets/fs117/en/

[2] Mohd-Zaki AH, Brett J, Ismail E, L'Azou M. Epidemiology of dengue disease in Malaysia (2000–2012): a systematic literature review. PLoS Negl Trop Dis. 2014 11 6;8(11):e3159.3. 25375211 10.1371/journal.pntd.0003159 PMC4222702

[3] Mudin RN. Dengue Incidence and the Prevention and Control Program in Malaysia. The International Medical Journal of Malaysia. 2015 6 1;14(1):05–10.

[4] Pun R, Shah Y, Gupta GP, Sherchand SP, Pandey BD. Prognostic value of rapid test for diagnosis of dengue in Nepalese patients during 2010 epidemic. Kathmandu University Medical Journal. 2012 10 2;10(1):3–6. 10.3126/kumj.v10i1.6905 22971853

[5] Ministry of Health Malaysia; Academy of Medicine Malaysia. Clinical practice guidelines: management of dengue infection in adults revised 2nd ed. Putrajaya: Ministry Of Health Malaysia; 2010.

[6] Grimshaw JM, Russell IT. Achieving health gain through clinical guidelines II: Ensuring guidelines change medical practice. Quality in health care. 1994 3;3(1):45–52. 10136260 10.1136/qshc.3.1.45 PMC1055182

[7] Saqib MA, Rafique I, Bashir S, Salam AA. A retrospective analysis of dengue fever case management and frequency of co-morbidities associated with deaths. BMC research notes. 2014 4 1;7(1):205. 24690140 10.1186/1756-0500-7-205 PMC3997840

[8] Afzal M, Tarar Sh, Akhtar M. Socio-demographic Characteristics and Clinical Spectrum of Dengue patients presenting to Aziz Bhatti Shaheed Teaching Hospital, Gujrat. Nature.;11:56–60.

[9] Chew MH, Rahman M, Salleh SA. Dengue in Malaysia: An epidemiological perspective study. Pakistan Journal of Medical Sciences. 2012 7 1;28(3):643–647.

[10] Ghosh G, Urhekar AD, Kosta S. A clinico-microbiological study of dengue fever cases in a tertiary care center of navi Mumbai. International Journal of Bioassays. 2013 10 31;2(11):1462–7.

[11] Ayyub M, Khazindar AM, Lubbad EH, Barlas S, Alfi AY, Al-Ukayli S. Characteristics of dengue fever in a large public hospital, Jeddah, Saudi Arabia. J Ayub Med Coll Abbottabad. 2006 4;18(2):9–13. 16977805

[12] Muhammad Azami NA, Azura Salleh S, Neoh HM, Syed Zakaria ZS, Jamal R. Dengue epidemic in Malaysia: Not a predominantly urban disease anymore. BMC Research Notes. 2011 12 1;4(1):1–4. 21714858 10.1186/1756-0500-4-216 PMC3154160

[13] Rowe EK, Leo YS, Wong JG, Thein TL, Gan VC, Lee LK, Lye DC. Challenges in dengue fever in the elderly: atypical presentation and risk of severe dengue and hospita-acquired infection. PLoS Negl Trop Dis. 2014 4 3;8(4):e2777 doi: 10.1371/journal.pntd.0002777 24699282 10.1371/journal.pntd.0002777 PMC3974675

[14] Khalil MA, Tan J, Khalil MA, Awan S, Rangasami M. Predictors of hospital stay and mortality in dengue virus infection-experience from Aga Khan University Hospital Pakistan. BMC research notes. 2014 7 27;7(1):1–7. 25064632 10.1186/1756-0500-7-473 PMC4115468

[15] World Health Organization, Special Programme for Research, Training in Tropical Diseases, World Health Organization. Department of Control of Neglected Tropical Diseases, World Health Organization. Epidemic, Pandemic Alert. Dengue: guidelines for diagnosis, treatment, prevention and control. World Health Organization; 2009.

