# Supplementary material for: Adherence to Clinical Practice Guidelines (CPG) management of dengue infection in adults (revised 2nd edition)
Source: PLoS One. 2017 Nov 2;12(11):e0184559. doi: 10.1371/journal.pone.0184559 (PMC5667811; doi:10.1371/journal.pone.0184559)
Supplement: S1 Appendix — (PDF) [file pone.0184559.s001.pdf]

S1 Appendix.

MINISTRY OF HEALTH
MALAYSIA

UNIVERSITY OF MALAYA

PRO-FORMA of MALAYSIAN CLINICAL PRACTICE GUIDELINE (CPG) ADHERENCE TO MANAGEMENT of DENGUE INFECTION IN ADULTS

INSTRUCTION: This questionnaire is divided into six (6) sections;

Part 1: Baseline
Part 2: Outpatient Department
Part 3: Emergency Department
Part 4: Medical Team
Part 5: Intensive Care Unit
Part 6: Overall outcome

Read the instruction carefully and please make sure all sections are filled with the right information. Thank you for your contribution in this project. Your contribution is highly appreciated.

Patient ID :			

BASELINE

Dengue CPG Adherence Pro-forma

Part I: Baseline data

1. Patient's Research ID			
2. Date of Birth (DOB)			
3. IC number / Passport No.			
4. Hospital Number/ RN			
5. Name of hospital			
6. Where was patient first seen? (Please circle)<br>1- Available<br>2- Not available	If available (Please circle)<br>1. Health Clinic<br>2. ED<br>3. Hospital Out-patient department (OPD)		
7. Date first seen at Health Facilities			
8. Date of Admission (dd/mm/yyyy)			
9. Date of Discharge (dd/mm/yyyy)			
10. Previous history of visit to private institution	Y=1,<br>a. Private clinic<br>b. Private hospital<br>N=2<br>NA=8		
11. Gender	female (1) / male (2)		
12. Body weight	…………………kg		
13. Height	…………………cm		
Co-morbidities	Yes	No	Remarks
14.Patients with co-morbidity<br>a. Diabetes			
b. Hypertension			
c. Ischaemic Heart Disease			
d. Coagulopathies			
e. Morbid Obesity			
f. Renal Failure			
g. Chronic Liver disease			
h. COPD			
i. Others (please specify)			
15. Previous history of dengue			
16. Elderly (more than 65 years old)			
17. Pregnancy			
18. Social factors that limit follow-up e.g.<br>a. living far from health facility			
b. no transport			
c. patient living alone			
19. Body Mass Index (BMI) kg/m^2			

20. Dengue Confirmatory test

	Done (Y=1,N=2,NA=8)			+ve result (Y=1,N=2,NA=8)		
	Yes	No	N/A	Yes	No	N/A
a. Dengue Ig G (if high titre, please specify)						
b. Dengue Ig M						
c. NS1 Ag						

Patient ID :			

OUT PATIENT DEPARTMENT

Part II: Stepwise Approach on OUT-PATIENT Management of Dengue Infection

VISIT :
DATE :
TIME :

A. History

	Documented		Please specify:
	Y=1	N=2	
1. Date of onset of fever			
a. Days of fever/illness			
2. Oral intake			
3. Diarrhea			
4. Bleeding			
5. Change in mental state/seizure/dizziness			
6. Urine output			
a. Frequency			
b. Volume			
c. Time of last voiding			
d. Others			

B. Assess for warning signs

	Documented		Please specify
	Y=1	N=2	
1. Abdominal pain or tenderness			
2. Persistent vomiting			
3. Clinical fluid accumulation (pleural effusion, ascites)			
1. Mucosal bleed			
2. Restlessness or lethargy			
3. Tender enlarged liver			
7. Laboratory : Increase in HCT concurrent with rapid decrease in platelet<br>* if any FBC available, considered yes			

C. Other important relevant histories

		Documented		Please specify
		Y=1	N=2	
1.	Family or neighborhood history of dengue			
2.	Jungle trekking and swimming in waterfall (consider leptospirosis, typhus, malaria)			
3.	Recent travel			
4.	Recent unprotected sexual or drug use behavior (consider acute HIV seroconversion illness)			

D. Physical examination

		Documented		Please specify
		Y=1	N=2	
1.	Assess mental state and Glasgow Coma Scale (GCS) score			
2.	Assess hydration status			
3.	Look out for tachypnea/ acidotic breathing			
4.	Look out for pleural effusion			
5.	Examine for bleeding manifestation			
6.	Check for abdominal tenderness			
7.	Check for hepatomegaly			
8.	Check for ascites			

E. Assess haemodynamic status

		Documented		Record value (if available)
		Y=1	N=2	
1.	Skin color			
2.	Cold/ warm extremities			
3.	Capillary filling time (normal <2 seconds)			
4.	Pulse rate			
5.	Pulse volume			
6.	Blood pressure			
7.	Pulse pressure			

F. Investigations

		Documented		Blood result
		Y=1	N=2	
**1.**	FBC			WBC:
				HB:
				PLT:
**2.**	HCT			
**3.**	Dengue serology/Dengue rapid test			

G. Diagnosis/Notification

		Tick (√) where appropriate	Please specify
1.	Phase of illness<br>a. Febrile		
	b. Deferversence / critical		
	c. Recovery		
2.	Dengue without warning sign		
3.	Dengue with warning sign		
4.	Severe dengue<br>a. Severe plasma leakage		
	b. Severe bleeding		
	c. Severe organ impairment		
5.	Notification within 24 hours from diagnosis		
6.	Outcome<br>a. Referral to hospital<br>b. Outpatient follow up		

H. Interval visit before admission.

1.Number of daily review	_______ days
2.Home Care Advice Leaflet for Dengue Patients given	Y = 1, N=2, NA=8

I. Prerequisites for transfer

	Documented		Please specify:		
	Y=1	N=2			
1. Patient was *optimized pretransfer					
2. ED/Medical was informed pretransfer					
3. Adequate Information includes fluid chart, monitoring chart and investigation Results given			Appropriate		Remarks
			Y=1	N=2	

*** optimize: complete resuscitation (stable VS)**

J. Referral from primary care providers to hospital

1. REFERRAL LETTER:
   a. Available= 1
   b. Not available= 2 (proceed to Part III if letter not available)

		Documented		Please specify
		Y=1	N=2	
2.	Symptoms :<br>a.Presence of 1 or more warning sign<br>***refer to section B**			
	b. Bleeding manifestations			
	c. Inability to tolerate oral fluids			
	d. Reduced urine output			
	e. Seizure			
3.	Signs :			
	a. Dehydration			
	b. Shock			
	c. Compensated shock			
	d. Bleeding			
	e. Any organ failure			
4.	Special Situations :			
	a. Patients with any of co-morbidity list :<br>i. Diabetes			
	ii. Hypertension			
	iii. Ischaemic Heart Disease			
	iv. Coagulopathies			
	v. Morbid Obesity			
	vi. Renal Failure			
	vii. Chronic Liver disease			
	viii. COPD			
	b. Elderly (more than 65 years old)			
	c. Pregnancy			
	d. Social factors that limit follow-up e.g. living far from health facility, no transport, patient living alone, etc			
5.	Laboratory Criteria:			
	a. Rising HCT with reducing platelet count *<br>(* minimum 1 result of FBC)			

Patient ID :			

Part III: ON ARRIVAL to hospital / EMERGENCY DEPARTMENT (data- admitting unit)

VISIT:
DATE:
TIME:
ZONE:

A. History

		Documented		Please specify
		Y=1	N=2	
1.	Date of onset of fever/ illness			
2.	Oral intake			
3.	Diarrhoea			
4.	Bleeding			
5.	Change in mental state/seizure/dizziness			
6.	Urine output			
	a. Frequency			
	b. Volume			
	c. Time of last voiding			

B. Assess for warning sign

		Documented		Please specify
		Y=1	N=2	
1.	Abdominal pain or tenderness			
2.	Persistent vomiting			
3.	Clinical fluid accumulation (pleural effusion, ascites)			
4.	Mucosal bleed			
5.	Restlessness or lethargy			
6.	Tender enlarged liver			
7.	Laboratory : Increase in HCT concurrent with rapid decrease in platelet			

C. Physical examination

		Documented		Please specify
		Y=1	N=2	
1.	Assess mental state and Glasgow Coma Scale (GCS) score			
2.	Assess hydration status			
3.	Look out for tachypnoea			
4.	Look out acidotic breathing			
5.	Look out for pleural effusion			
6.	Examine for bleeding manifestation			
7.	Check for abdominal tenderness			
8.	Check for hepatomegaly			
9.	Check for ascites			

D. Assess haemodynamic status

		Done		Value	Remarks
		Y=1	N=2		
1.	Skin color				
2.	Cold/ warm extremities				
3.	Capillary filling time (normal <2 seconds)				
4.	Pulse rate				
5.	Pulse volume				
6.	Blood pressure				
7.	Pulse pressure				

E. Diagnosis /Notification

		Documented		Please specify
		Y=1	N=2	
1.	Phase of illness<br>a. Febrile			
	b. Deferversence			
	c. recovery			
2.	Dengue without warning sign			
3.	Dengue with warning sign			
4.	Severe dengue<br>a. severe plasma leakage			
	b. severe bleeding			
	c. severe organ impairment			
5.	Notification within 24 hours from diagnosis			

F. Initial management ED (follow Table 8 from CPG) – Please tick at Table 8 whenever appropriate

		Documented (Y=1,N=2)					
		Febrile		Critical		Recovery	
1.		Yes	No	Yes	No	Yes	No
	Investigation						
	a. FBC & HCT						
	b. Dengue Serology						
2.	Monitoring						
	a. Pink/ cyanosis						
	b. Extremities (cold/warm)						
	c. Capillary refill time						
	d. Pulse volume						
	e. Pulse rate						
	f. Blood pressure						
	g. Pulse pressure						
	h. Respiratory rate						
	i.SpO2						
	j. warning sign assessment						
	k. urine output						
3.	Fluid management						
	a. Bolus 20ml/kg						
	b. Half bolus 10ml/kg						
	c. 7 ml/kg regime						
	d. 5 ml/kg regime						
	e. 3 ml/kg regime						
	f. 2 ml/kg regime						
	g. Blood transfusion						

G. Follow through plan in ED

		Tick (√) where appropriate	Please specify
1.	Discharge without follow up		
2.	Discharge with follow up		
3.	Direct Admission		
4.	Medical referral		
	a. Discharge		
	b. Admit		
5.	ICU referral		
6.	Death		
1.	Complication		

Table 8 : Parameters and Frequency of Monitoring According to Different Phases of Dengue Illness

Parameters for monitoring	Frequency of monitoring		
	Febrile phase	Critical phase	Recovery phase
**Clinical Parameters**			
**General well being**<br>**Appetite/ oral intake**<br>**Warning signs**<br>**Symptoms of bleeding**<br>**Neurological/ mental state**	Daily or more frequently towards late febrile phase	At least twice a day and more frequently as indicated	Daily or more frequently as indicated
**Haemodynamic status**<br>• *Pink/ cyanosis*<br>• *Extremities (cold/warm)*<br>• *Capillary refill time*<br>• *Pulse volume*<br>• *PR*<br>• *BP*<br>• *Pulse pressure*<br><br>**Respiratory status**<br>• *RR*<br>• *SpO_2*	4-6 hourly depending on clinical status	2-4 hourly depending on clinical status<br><br>**In shock**<br>Every 15-30 minutes till stable then 1-2 hourly	4-6 hourly
**Signs of bleeding, abdominal tenderness, ascites and pleural effusion**	Daily or more frequently towards late febrile phase	At least twice a day and more frequently as indicated	Daily or more frequently as indicated
**Urine output**	4 hourly	2-4 hourly<br>**In shock**<br>Hourly	4-6 hourly
**Parameters for monitoring**	**Frequency of monitoring**		
	**Febrile phase**	**Critical phase**	**Recovery phase**
**Clinical Parameters**			
**FBC + HCT**	Daily or more frequently if indicated	4-12 hourly depending on clinical status<br>**In shock**<br>Repeated before and after each attempt of fluid resuscitation and as indicated	Daily
**BUSE/ Creatinine**<br>**LFT**<br>**RBS**<br>**Coagulation profile**<br>**HCO_3 / TCO_2 / Lactate**	As indicated	At least daily or more frequently as indicated<br>**In shock**<br>Crucial to monitor acid-base balance/ ABG closely	As indicated

Adapted from [2, Level 9; 65, Level 9]

Patient ID :			

MEDICAL TEAM

Part IV: IN-PATIENT Management of Dengue infection (MEDICAL TEAM)

DATE:

A. History (FIRST REVIEW)

		Documented		Please specify
		Y=1	N=2	
1.	Date of onset of fever/ illness			
2.	Oral intake			
3.	Diarrhea			
4.	Bleeding			
5.	Change in mental state/seizure/dizziness			
6.	Urine output (frequency, volume and time of last voiding)			

B. Assess for warning sign (FIRST REVIEW)

		Documented		Please specify
		Y=1	N=2	
1.	Abdominal pain or tenderness			
2.	Persistent vomiting			
3.	Clinical fluid accumulation (pleural effusion, ascites)			
4.	Mucosal bleed			
5.	Restlessness or lethargy			
6.	Tender enlarged liver			
7.	Laboratory : Increase in HCT concurrent with rapid decrease in platelet			

C. Physical examination (FIRST REVIEW)

		Documented		Please specify
		Y=1	N=2	
1.	Assess mental state and Glasgow Coma Scale (GCS) score			
2.	Assess hydration status			
3.	Look out for tachypnea/RR / Look out acidotic breathing			
4.	Look out for pleural effusion			
5.	Examine for bleeding manifestation			
6.	Check for abdominal tenderness			
7.	Check for hepatomegaly			
8.	Check for ascites			

D. Assess haemodynamic status (FIRST REVIEW)

		Documented		Value	Remarks
		Y=1	N=2		
1.	Skin color				
2.	Cold/ warm extremities				
3.	Capillary filling time (normal <2 seconds)				
4.	Pulse rate				
5.	Pulse volume				
6.	Blood pressure				
7.	Pulse pressure				

E. Diagnosis / Notification (FIRST REVIEW)

		Done		Please specify:
		Y=1	N=2	
1.	**Phase of illness**<br>a. Febrile			
	b. Deferversence			
	c. recovery			
2.	Dengue without warning sign			
3.	Dengue with warning sign			
4.	Severe dengue<br>a. Severe plasma leakage			
	b. Severe bleeding			
	c. Severe organ impairment			
5.	Notification done within 24 hours			

F. Ward management (follow Table 8)

		Documented (Y=1,N=2)					
		Febrile		Critical		Recovery	
1.		Yes	No	Yes	No	Yes	No
	Investigation						
	a. FBC & HCT						
	b. Dengue serology						
2.	Monitoring						
	a. Pink/ cyanosis						
	b. Extremities (cold/warm)						
	c. Capillary refill time						
	d. Pulse volume						
	e. PR						
	f. BP						
	g. Pulse pressure						
	h. RR						
	i. SpO2						
	j. Warning sign assessment						
	k. Urine output						

Table 8 : Parameters and Frequency of Monitoring According to Different Phases of Dengue Illness

Parameters for monitoring	Frequency of monitoring		
	Febrile phase	Critical phase	Recovery phase
**Clinical Parameters**			
**General well being**<br>**Appetite/ oral intake**<br>**Warning signs**<br>**Symptoms of bleeding**<br>**Neurological/ mental state**	Daily or more frequently towards late febrile phase	At least twice a day and more frequently as indicated	Daily or more frequently as indicated
**Haemodynamic status**<br>• *Pink/ cyanosis*<br>• *Extremities (cold/warm)*<br>• *Capillary refill time*<br>• *Pulse volume*<br>• *PR*<br>• *BP*<br>• *Pulse pressure*<br><br>**Respiratory status**<br>• *RR*<br>• *SpO_2*	4-6 hourly depending on clinical status	2-4 hourly depending on clinical status<br><br>**In shock**<br>Every 15-30 minutes till stable then 1-2 hourly	4-6 hourly
**Signs of bleeding, abdominal tenderness, ascites and pleural effusion**	Daily or more frequently towards late febrile phase	At least twice a day and more frequently as indicated	Daily or more frequently as indicated
**Urine output**	4 hourly	2-4 hourly<br>**In shock**<br>Hourly	4-6 hourly
**Parameters for monitoring**	**Frequency of monitoring**		
	**Febrile phase**	**Critical phase**	**Recovery phase**
**Clinical Parameters**			
**FBC + HCT**	Daily or more frequently if indicated	4-12 hourly depending on clinical status<br>**In shock**<br>Repeated before and after each attempt of fluid resuscitation and as indicated	Daily
**BUSE/ Creatinine**<br>**LFT**<br>**RBS**<br>**Coagulation profile**<br>**HCO_3/ TCO_2/ Lactate**	As indicated	At least daily or more frequently as indicated<br>**In shock**<br>Crucial to monitor acid-base balance/ ABG closely	As indicated

Adapted from [2, Level 9; 65, Level 9]

G. Overall fluid management (Tick where appropriate)

		DF + w/out WS	DF + WS	DF Compensated Shock	DF Decompensated Shock	Severe Dengue
a.	bolus 20ml/kg					
b.	half bolus 10ml/kg					
c.	7 ml/kg regime					
d.	5 ml/kg regime					
e.	3 ml/kg regime					
f.	2 ml/kg regime					
g.	mantainence					
h.	blood transfusion					
i.	other regime					

H. Follow through plan by Medical Team

		Tick (√) where appropriate	Please specify
1.	Discharge without follow up		
2.	Discharge with follow up		
4.	Transfer to ICU (Please complete part I)		
5.	Care by medical team		
6.	Death		
7.	Complication:		
	a. Thrombophlebitis		
	b. Fluid overload		
	c. Hospital Acquired Pneumonia		
	d. Other		

I. PRE TRANSFER TO INTENSIVE CARE UNIT ASSESSMENT(when applicable)

(i) History (PRE ICU TRANSFER)

		Documented		Please specify
		Y=1	N=2	
1.	Date of onset of fever/ illness			
2.	Oral intake			
3.	Diarrhea			
4.	Bleeding			
5.	Change in mental state/seizure/dizziness			
6.	Urine output (frequency, volume and time of last voiding)			

(ii) Assess for warning sign (PRE ICU TRANSFER)

		Documented		Please specify
		Y=1	N=2	
1.	Abdominal pain or tenderness			
2.	Persistent vomiting			
3.	Clinical fluid accumulation (pleural effusion, ascites)			
4.	Mucosal bleed			
5.	Restlessness or lethargy			
6.	Tender enlarged liver			
7.	Laboratory : Increase in HCT concurrent with rapid decrease in platelet			

(iii) Physical examination (PRE ICU TRANSFER)

		Documented		Please specify
		Y=1	N=2	
1.	Assess mental state and Glasgow Coma Scale (GCS) score			
2.	Assess hydration status			
3.	Look out for tachypnea/RR / Look out acidotic breathing			
4.	Look out for pleural effusion			
5.	Examine for bleeding manifestation			
6.	Check for abdominal tenderness			
7.	Check for hepatomegaly			
8.	Check for ascites			

(iv) Assess haemodynamic status (PRE ICU TRANSFER)

		Documented		Value
		Y=1	N=2	
1.	Skin color			
2.	Cold/ warm extremities			
3.	Capillary filling time (normal <2 seconds)			
4.	Pulse rate			
5.	Pulse volume			
6.	Blood pressure			
7.	Pulse pressure			

(v) Diagnosis (PRE ICU TRANSFER)

		Done		Please specify:
		Y=1	N=2	
1.	Phase of illness<br>a. Febrile			
	b. Deferversence			
	c. Recovery			
2.	Dengue without warning sign			
3.	Dengue with warning sign			
4.	Severe dengue<br>a. severe plasma leakage			
	b. severe bleeding			
	c. severe organ impairment			
5.	Notification done within 24 hours			

(vi) PRE ICU TRANSFER management (follow Table 8)

		Documented (Y=1,N=2)					
		Febrile		Critical		Recovery	
1.		Yes	No	Yes	No	Yes	No
	Investigation						
	a. FBC & HCT						
	b. Dengue serology						
2.	Monitoring						
	a. Pink/ cyanosis						
	b. Extremities (cold/warm)						
	c. Capillary refill time						
	d. Pulse volume						
	e.PR						
	f. BP						
	g. Pulse pressure						
	h. RR						
	i.SpO2						
	j. warning sign assessment						
	k. urine output						

(vii) Overall fluid management (PRE ICU) (Tick where appropriate)

		**DF + w/out WS**	**DF + WS**	**DF compensated Shock**	**DF decompensated shock**	**Severe Dengue**
a.	bolus 20ml/kg					
b.	half bolus 10ml/kg					
c.	7 ml/kg regime					
d.	5 ml/kg regime					
e.	3 ml/kg regime					
f.	2 ml/kg regime					
g.	mantainence					
h.	blood transfusion					
i.	other regime					

Table 8 : Parameters and Frequency of Monitoring According to Different Phases of Dengue Illness

Parameters for monitoring	Frequency of monitoring		
	**Febrile phase**	**Critical phase**	**Recovery phase**
**Clinical Parameters**			
**General well being**<br>**Appetite/ oral intake**<br>**Warning signs**<br>**Symptoms of bleeding**<br>**Neurological/ mental state**	Daily or more frequently towards late febrile phase	At least twice a day and more frequently as indicated	Daily or more frequently as indicated
**Haemodynamic status**<br>• *Pink/ cyanosis*<br>• *Extremities (cold/warm)*<br>• *Capillary refill time*<br>• *Pulse volume*<br>• *PR*<br>• *BP*<br>• *Pulse pressure*<br><br>**Respiratory status**<br>• *RR*<br>• *SpO_2*	4-6 hourly depending on clinical status	2-4 hourly depending on clinical status<br><br>**In shock**<br>Every 15-30 minutes till stable then 1-2 hourly	4-6 hourly
**Signs of bleeding, abdominal tenderness, ascites and pleural effusion**	Daily or more frequently towards late febrile phase	At least twice a day and more frequently as indicated	Daily or more frequently as indicated
**Urine output**	4 hourly	2-4 hourly<br>**In shock**<br>Hourly	4-6 hourly
**Parameters for monitoring**	**Frequency of monitoring**		
	**Febrile phase**	**Critical phase**	**Recovery phase**
**Clinical Parameters**			
**FBC + HCT**	Daily or more frequently if indicated	4-12 hourly depending on clinical status<br>**In shock**<br>Repeated before and after each attempt of fluid resuscitation and as indicated	Daily
**BUSE/ Creatinine**<br>**LFT**<br>**RBS**<br>**Coagulation profile**<br>**HCO_3/ TCO_2/ Lactate**	As indicated	At least daily or more frequently as indicated<br>**In shock**<br>Crucial to monitor acid-base balance/ ABG closely	As indicated

Adapted from [2, Level 9; 65, Level 9]

Patient ID :			

Part V: IN-PATIENT Management of Dengue infection by ICU TEAM (when applicable)

DATE:

A. History (FIRST REVIEW)

		Documented		Please specify
		Y=1	N=2	
1.	Date of onset of fever/ illness			
2.	Oral intake			
3.	Diarrhea			
4.	Bleeding			
5.	Change in mental state/seizure/dizziness			
6.	Urine output (frequency, volume and time of last voiding)			

B. Assess for warning sign (FIRST REVIEW)

		Documented		Please specify
		Y=1	N=2	
1.	Abdominal pain or tenderness			
2.	Persistent vomiting			
3.	Clinical fluid accumulation (pleural effusion, ascites)			
4.	Mucosal bleed			
5.	Restlessness or lethargy			
6.	Tender enlarged liver			
7.	Laboratory : Increase in HCT concurrent with rapid decrease in platelet			

C. Physical examination (FIRST REVIEW)

		Documented		Please specify
		Y=1	N=2	
1.	Assess mental state and Glasgow Coma Scale (GCS) score			
2.	Assess hydration status			
3.	Look out for tachypnea/RR / Look out acidotic breathing			
4.	Look out for pleural effusion			
5.	Examine for bleeding manifestation			
6.	Check for abdominal tenderness			
7.	Check for hepatomegaly			
8.	Check for ascites			

D. Assess haemodynamic status (FIRST REVIEW)

		Documented		Value
		Y=1	N=2	
1.	Skin color			
2.	Cold/ warm extremities			
3.	Capillary filling time (normal <2 seconds)			
4.	Pulse rate			
5.	Pulse volume			
6.	Blood pressure			
7.	Pulse pressure			

E. Diagnosis / Notification (FIRST REVIEW)

		Done		Please specify
		Y=1	N=2	
1.	Phase of illness<br>a. Febrile<br>b. Deferversence<br>c. recovery			
2.	Dengue without warning sign			
3.	Dengue with warning sign			
4.	Severe dengue<br>a. severe plasma leakage<br>b. severe bleeding<br>c. severe organ impairment			
5.	Notification done within 24 hours			

F. Ward management (follow Table 8)

		Documented (Y=1,N=2)					
		Febrile		Critical		Recovery	
1.		Yes	No	Yes	No	Yes	No
	Investigation						
	a. FBC & HCT						
	b. Dengue serology						
2.	Monitoring						
	a. Pink/ cyanosis						
	b. Extremities (cold/warm)						
	c. Capillary refill time						
	d. Pulse volume						
	e. PR						
	f. BP						
	g. Pulse pressure						
	h. RR						
	i. SpO2						
	j. warning sign assessment						
	k. urine output						

G. Overall fluid management (Tick where appropriate)

	DF + w/out WS	DF + WS	DF compensated Shock	DF decompensated shock	Severe Dengue
a. bolus 20ml/kg					
b. half bolus 10ml/kg					
c. 7 ml/kg regime					
d. 5 ml/kg regime					
e. 3 ml/kg regime					
f. 2 ml/kg regime					
g. mantainence					
h. blood transfusion					
i. other regime					

H. Follow through plan by ICU Team

		Tick (√) where appropriate	Please specify
4.	Transfer to Medical team (please complete part I)		
5.	Transfer to other team		
6.	Death		
7.	Complication:		
	a. Thrombophlebitis		
	b. Fluid overload		
	c. Hospital Acquired Pneumonia		
	d. Other		

Table 8 : Parameters and Frequency of Monitoring According to Different Phases of Dengue Illness

Parameters for monitoring	Frequency of monitoring		
	Febrile phase	Critical phase	Recovery phase
**Clinical Parameters**			
**General well being**<br>**Appetite/ oral intake**<br>**Warning signs**<br>**Symptoms of bleeding**<br>**Neurological/ mental state**	Daily or more frequently towards late febrile phase	At least twice a day and more frequently as indicated	Daily or more frequently as indicated
**Haemodynamic status**<br>• *Pink/ cyanosis*<br>• *Extremities (cold/warm)*<br>• *Capillary refill time*<br>• *Pulse volume*<br>• *PR*<br>• *BP*<br>• *Pulse pressure*<br><br>**Respiratory status**<br>• *RR*<br>• *SpO_2*	4-6 hourly depending on clinical status	2-4 hourly depending on clinical status<br><br>**In shock**<br>Every 15-30 minutes till stable then 1-2 hourly	4-6 hourly
**Signs of bleeding, abdominal tenderness, ascites and pleural effusion**	Daily or more frequently towards late febrile phase	At least twice a day and more frequently as indicated	Daily or more frequently as indicated
**Urine output**	4 hourly	2-4 hourly<br>**In shock**<br>Hourly	4-6 hourly
**Parameters for monitoring**	**Frequency of monitoring**		
	**Febrile phase**	**Critical phase**	**Recovery phase**
**Clinical Parameters**			
**FBC + HCT**	Daily or more frequently if indicated	4-12 hourly depending on clinical status<br>**In shock**<br>Repeated before and after each attempt of fluid resuscitation and as indicated	Daily
**BUSE/ Creatinine**<br>**LFT**<br>**RBS**<br>**Coagulation profile**<br>**HCO_3/ TCO_2/ Lactate**	As indicated	At least daily or more frequently as indicated<br>**In shock**<br>Crucial to monitor acid-base balance/ ABG closely	As indicated

Adapted from [2, Level 9; 65, Level 9]

I. PRE TRANSFER BACK TO MEDICAL WARD

(i) History (PRE TRANSFER BACK TO MEDICAL)

		Documented		Please specify
		Y=1	N=2	
1.	Date of onset of fever/ illness			
2.	Oral intake			
3.	Diarrhea			
4.	Bleeding			
5.	Change in mental state/seizure/dizziness			
6.	Urine output (frequency, volume and time of last voiding)			

(ii) Assess for warning sign(PRE TRANSFER BACK TO MEDICAL)

		Documented		Please specify
		Y=1	N=2	
1.	Abdominal pain or tenderness			
2.	Persistent vomiting			
3.	Clinical fluid accumulation (pleural effusion, ascites)			
4.	Mucosal bleed			
5.	Restlessness or lethargy			
6.	Tender enlarged liver			
7.	Laboratory : Increase in HCT concurrent with rapid decrease in platelet			

(iii) Physical examination(PRE TRANSFER BACK TO MEDICAL)

		Documented		Please specify
		Y=1	N=2	
1.	Assess mental state and Glasgow Coma Scale (GCS) score			
2.	Assess hydration status			
3.	Look out for tachypnea/RR / Look out acidotic breathing			
4.	Look out for pleural effusion			
5.	Examine for bleeding manifestation			
6.	Check for abdominal tenderness			
7.	Check for hepatomegaly			
8.	Check for ascites			

(iv) ***Assess haemodynamic status(PRE TRANSFER BACK TO MEDICAL)***

		Documented		Value
		Y=1	N=2	
1.	Skin colour			
2.	Cold/ warm extremities			
3.	Capillary filling time (normal <2 seconds)			
4.	Pulse rate			
5.	Pulse volume			
6.	Blood pressure			
7.	Pulse pressure			

(v) ***Diagnosis(PRE TRANSFER BACK TO MEDICAL)***

		Done		Please specify:
		Y=1	N=2	
1.	Phase of illness<br>a. Febrile			
	b. Deferversence			
	c. recovery			
2.	Dengue without warning sign			
3.	Dengue with warning sign			
4.	Severe dengue<br>a. severe plasma leakage			
	b. severe bleeding			
	c. severe organ impairment			
5.	Notification done within 24 hours			

(vi) ***PRE TRANSFER BACK TO MEDICAL management (follow Table* 8)**

		Documented (Y=1,N=2)					
		Febrile		Critical		Recovery	
1.		**Yes**	**No**	**Yes**	**No**	**Yes**	**No**
	Investigation						
	a. FBC & HCT						
	b. Dengue serology						
2.	Monitoring						
	a. Pink/ cyanosis						
	b. Extremities (cold/warm)						
	c. Capillary refill time						
	d. Pulse volume						
	e. PR						
	f. BP						
	g. Pulse pressure						
	h. RR						
	i. SpO2						
	j. warning sign assessment						
	k. urine output						

Table 8 : Parameters and Frequency of Monitoring According to Different Phases of Dengue Illness

Parameters for monitoring	Frequency of monitoring		
	Febrile phase	Critical phase	Recovery phase
**Clinical Parameters**			
**General well being**<br>**Appetite/ oral intake**<br>**Warning signs**<br>**Symptoms of bleeding**<br>**Neurological/ mental state**	Daily or more frequently towards late febrile phase	At least twice a day and more frequently as indicated	Daily or more frequently as indicated
**Haemodynamic status**<br>• *Pink/ cyanosis*<br>• *Extremities (cold/warm)*<br>• *Capillary refill time*<br>• *Pulse volume*<br>• *PR*<br>• *BP*<br>• *Pulse pressure*<br><br>**Respiratory status**<br>• *RR*<br>• *SpO_2*	4-6 hourly depending on clinical status	2-4 hourly depending on clinical status<br><br>**In shock**<br>Every 15-30 minutes till stable then 1-2 hourly	4-6 hourly
**Signs of bleeding, abdominal tenderness, ascites and pleural effusion**	Daily or more frequently towards late febrile phase	At least twice a day and more frequently as indicated	Daily or more frequently as indicated
**Urine output**	4 hourly	2-4 hourly<br>**In shock**<br>Hourly	4-6 hourly
**Parameters for monitoring**	**Frequency of monitoring**		
	**Febrile phase**	**Critical phase**	**Recovery phase**
**Clinical Parameters**			
**FBC + HCT**	Daily or more frequently if indicated	4-12 hourly depending on clinical status<br>**In shock**<br>Repeated before and after each attempt of fluid resuscitation and as indicated	Daily
**BUSE/ Creatinine**<br>**LFT**<br>**RBS**<br>**Coagulation profile**<br>**HCO_3/ TCO_2/ Lactate**	As indicated	At least daily or more frequently as indicated<br>**In shock**<br>Crucial to monitor acid-base balance/ ABG closely	As indicated

Adapted from [2, Level 9; 65, Level 9]

(vii) ***Overall fluid management (PRE TRANSFER BACK TO MEDICAL)***

	**DF + w/out WS**	**DF + WS**	**DF compensated Shock**	**DF decompensated shock**	**Severe Dengue**
a. bolus 20ml/kg					
b. half bolus 10ml/kg					
c. 7 ml/kg regime					
d. 5 ml/kg regime					
e.3 ml/kg regime					
f. 2 ml/kg regime					
g. mantainence					
h. blood transfusion					
i.other regime					

Patient ID :			

Part VI: Overall outcome of this dengue episode

A. Patient outcome

		Tick (√) where appropriate
1.	Discharge without follow up	
2.	Discharge with follow up	
3.	ICU referral	
	a. Admit	
	b. Non-invasive Ventilation	
	c. Invasive Ventilation	
4.	Complication	
	a. Thrombophlebitis	
	b. Fluid overload	
	c. Hospital Acquired Pneumonia	
	d. Other	
5.	Death	
6.	Total length of hospital stay	_________ day/s

B. Discharge Criteria:

**Criteria**	**Documented**		**Please specify**
	**Y=1**	**N=2**	
Afebrile for 48H			
Improved general condition			
Improved appetite			
Stable haematocrit			
Rising platelet count			
No dyspnea or respiratory distress			
Resolved bleeding episodes			
Resolutions/recovery of organ dysfunction			
